# Public-private options for expanding access to human resources for HIV/AIDS in Botswana

**DOI:** 10.1186/1478-4491-5-25

**Published:** 2007-10-19

**Authors:** Norbert Dreesch, Jennifer Nyoni, Ontlametse Mokopakgosi, Khumo Seipone, Jean Alfazema Kalilani, Owen Kaluwa, Vincent Musowe

**Affiliations:** 1 Human Resources for Health, World Health Organization, Geneva, Switzerland; 2 Human Resources for Health Management, World Health Organization, Regional Office for Africa, Brazzaville, Congo; 3 Policy, Planning, Monitoring and Evaluation, Ministry of Health, Gaborone, Botswana; 4 Department of HIV/AIDS Prevention and Care, Ministry of Health, Gaborone, Botswana; 5 World Health Organization, Botswana; 6 HIV, World Health Organization, Botswana; 7 Ministry of Health, Zambia

## Abstract

In responding to the goal of rapidly increasing access to antiretroviral treatment (ART), the government of Botswana undertook a major review of its health systems options to increase access to human resources, one of the major bottlenecks preventing people from receiving treatment. In mid-2004, a team of government and World Health Organization (WHO) staff reviewed the situation and identified a number of public sector scale up options. The team also reviewed the capacity of private practitioners to participate in the provision of ART. Subsequently, the government created a mechanism to include private practitioners in rolling out ART. At the end of 2006, more than 4500 patients had been transferred to the private sector for routine follow up. It is estimated that the cooperation reduced the immediate need for recruiting up to 40 medically qualified staff into the public sector over the coming years, depending on the development of the national standard for the number and duration of patient visits to a doctor per year. Thus welcome relief was brought, while at the same time not exercising a pull factor on human resources for health in the sub-Saharan region.

## Review

Botswana is a sub-Saharan African country in which the HIV prevalence is among the highest in the world (see Table 1). The epidemic reached mature proportions over the past decade, with deaths from the disease affecting all strata of society. Given the alarming prevalence figures reported during the 1990s, presidential level commitment assured a strong response to the expanding epidemic throughout Botswana society by creating the National AIDS Coordinating Agency in 2002.

**Table 1:** Country background data [10,11,12]

Total population	1 765 000	(2004)
GNI per capita (International $)	8 920	(2004)
Under- five mortality m/f (per 1000)	116	(2004)
Adult mortality m/f (per 1000)	786/710	(2004)
Total health expenditure per capita (International $)	375	(2004)
Total health expenditure as % of GDP	5.6	(2004)
National HIV prevalence	17.10%	(2004)
Number HIV positive women (15–49)	157 783	(2005) (Sentinel survey data)
Number HIV positive men (15–49)	98 423	(2005) (Sentinel survey data)

As of the mid-1990s, ART became increasingly affordable globally. As a middle-income country, Botswana decided with the highest political commitment to invest heavily through creating the ART Programme to address the need to provide access to antiretroviral therapy. In 2000 the government contracted the McKinsey consulting company who conducted a situation assessment for the establishment of the ART Program. In addition, they provided an analysis of treatment needs and a way to organize the ART programme. The target coverage of need for ART and care was established at 110 000 patients as a result of this consultancy [1].

As an initial emergency response, a public-sector antiretroviral programme was created to focus support on delivery of this specific component of HIV/AIDS prevention, care and treatment services.

In mid-2004, this programme had evolved to consist of specialized antiretroviral therapy public clinics established in existing health care facilities and operating in four sites across the country. A site consists of one hospital with up to four satellite clinics. At that time, the population response to the need for testing for HIV was such that 10 000 patients who had tested positive and were found to be in need of ART could not start therapy due to insufficient access to the health workforce. Thus, there was a need to identify other health system resources to provide access to human resources for ART.

In the meantime, many other donors and bilateral aid agencies, including the Bill & Melinda Gates Foundation, Baylor College of Medicine and Harvard University, CDC-Botswana, Bristol-Myers Squibb, the African Comprehensive HIV/AIDS Partnerships (ACHAP) had all become active collaborators in teaching, training, setting up laboratory facilities or designing a roll-out programme for antiretroviral care. Likewise, the World Health Organization (WHO) provided technical assistance to set up the programme by fielding an officer to support the creation and roll-out of a training programme called KITSO ('Knowledge, Innovation, and Training Shall Overcome AIDS', KITSO is the Setswana word for 'knowledge').

### Increasing international support and national response

One of the priorities of the WHO Director-General, the late Dr LEE Jong-wook, who took office in July 2003, was increasing access to anti-retroviral care. This led to the announcement of the goal of reaching half of the 6 million in need globally with access to treatment at the end of 2005. This was announced on World AIDS Day in December 2003 [2].

Given the impetus of the '3 by 5' initiative starting in 2004, serving those waiting for access to care was critical in a number of ways in Botswana:

• avoiding unnecessary suffering and premature deaths;

• maintaining the momentum of population response to come forward for testing by offering rapid access to treatment in case of need;

• avoiding public discontent with service provision and possible negative consequences for the uptake of HIV testing;

• increasing morale levels among staff, who could now see that treatment was possible but should not be allowed to become frustrated by the continuing lack of access.

The ensuing WHO technical support in mid-2004 brought together a multidisciplinary team from the Ministry of Health and WHO to address the issues of increasing access to human resources within the current health system, with a particular focus on HIV/AIDS care services.

### The health workforce situation in Botswana in 2004

With the exception of nursing and some other non-medical staff, Botswana relies heavily on importing human resources, particularly doctors, both generalists and specialists. The country does not as yet have its own medical school to respond to its medical workforce needs, although plans are under way to add a medical school to the existing university departments. Table 2 provides an overview of the human resources situation in the country in 2004.

**Table 2:** National human resources in Botswana, 2004 [13]

**Type/category**	**Established posts**	**Filled posts**	**Vacant posts**
Medical specialists	182	165	17
Medical officers	407	370	37
Nurses	4753	4583	170
Pharmacists	145	140	5
Pharmacy technicians	211	193	18
Lab scientists	38	35	3
Lab technicians	167	160	7
Lab Assistants	23	21	2
Dieticians/nutritionists	24	19	5
Radiographers/radiology officers	64	61	3
Family Welfare Educators (FWEs)	981	829	152
Health education officers	50	48	2
Lay counsellors	302	300	2
Social workers	96	96	0

To roll out the antiretroviral therapy strategy throughout the country, a number of additional positions were authorized in the public sector to allow for phased implementation. Table 3 presents the positions approved to start scale-up in 2004. These contract positions attracted personnel from the Southern African Development Community (SADC) region.

**Table 3:** Positions approved to start the PMTCT and ART programme [14]

**Category**	**Established**	**Filled**	**Vacant**
Medical specialists*	2	2	0
Medical officers	38	38	0
Nurses	129	127	2
Pharmacists	10	10	0
Pharmacy technicians	37	37	0
Lab technicians	17	16	1
Dieticians/nutritionists	2	2	0
Social workers	28	28	0
Lay counsellors	302	300	2

The core team needed to start providing ART services was identified as presented in Table 4. The core support team at satellite clinics and its composition to start treatment is depicted in Table 5 and patient enrolment targets had been set as indicated in Table 6.

**Table 4:** Core treatment team composition in the hospital, minimum numbers to be placed to start treatment [14]

Secondment doctor	1
Physician/Medical officer	2
Paediatrician	1
Nurse	3
Phlebotomist (lab tech or retrained nurse)	2
Counsellors	3
Dietician/nutritionist	1
Administrative/data entry clerk	1
Pharmacist	2
Pharmacy technician	2
*Total*	*18*

**Table 5:** Core support team composition at clinics, minimum numbers to be placed to start treatment [14]

Medical officer	1
Nurse	1
Counsellor (social worker)	1
Pharmacy technician/pharmacist	1
Laboratory technician	1
Home-based care volunteers	
Family Welfare Educators (FWEs)	
Total	5

**Table 6:** Patient year-end enrolment targets, 2003 – 2005 [14]

2003	15 000
2004	25 000–30 000 (21 000 as of May 2004)
2005	45 000–50 000

### Challenges and opportunities for scaling up access to human resources

In 2004, approximately 21 000 persons were on treatment and 10 000 whose CD4 cell count had been established were awaiting access to treatment [3]. The treatment goal of the 3 by 5 initiative was to reach half of the target of 110 000 people in need of ART by December 2005. Thus, by mid-2004 another 34 000 more persons needed to be provided with access to treatment during the course of the next 1.5 years.

Given that the available health workforce was providing ART to 21 000 persons (approximately 1/3 private, 2/3 public sector human resources/ART and facility/equipment access), the challenge of meeting the needs of an additional 34 000 patients would correlate to additional workforce needs.

Given the model of care at the time, the ART programme had estimated the need for another 40 doctors for the 3 by 5 goal to be met, which would roughly translate into a patient load of 850 patients per doctor per year. This figure seemed to be in line with findings from other recent studies of ART treatment sites and workforce tasks and skills use [4,5]. It should be noted, however, that great variability in the need for coverage and physician skills was also reported in these studies, depending on the distribution of tasks between the various categories in the care team [4,5]. Given the difficulties likely to be encountered in recruitment drives that would need to yield almost immediate results, further local analyses were needed to identify ways to provide care to more patients more efficiently. This could include simplification of current ART modalities, updating and transfer of skills, integration of an ART package into in- and pre-service training modules, diversification of training programmes to achieve a 3 by 5 responsive skill mix and the possible redistribution of tasks within the treatment team.

### The challenge of ART providers and their potential

Many institutions from the public and private sector, civil society, community-based organizations and organizations of persons living with HIV/AIDS (PLWA) contribute to the fight against HIV/AIDS in Botswana. In addition to government public health services, the key institutions involved in the delivery of ART services include mission hospitals, the Botswana Defence Force (BDF), the Botswana Meat Commission (BMC), the Debswana treatment initiatives operating at Orapa and Jwaneng and the Bamangwato Concessions Limited (BCL). Development partners and NGO initiatives, including networks of PLWA all support the ART program in one way or another. The ART program has linkages with other programmes such tuberculosis (TB), preventing mother-to-child transmission (PMTCT), testing and counselling, and community home-based care and all these interact to increase access of the population to essential elements of a continuum of care in HIV/AIDS.

As health programmes with specific goals and targets mature, more experience is gained on ways to further strengthen programme effectiveness and efficiency. In terms of health workforce use and deployment, skills development and skills distribution among staff, mature programmes tend to identify ways to derive synergies from a certain degree of integration. Therefore almost all programmes provided through the primary health care networks have the potential to contribute substantially to access to ART as well as to prevention and support.

### Potential contribution of simplified access and patient flows to skilled ART staff

At the inception of the ART and PMTCT Programme, additional health staff were recruited to support the ART and PMTCT programme implementation. Expansion of rapid access to ART depends on who can initiate treatment and administer drugs. At the time, the project document required that a medical officer initiate the treatment cycle and that only the pharmacist could dispense antiretrovirals. Given the shortage of medically qualified personnel, it was necessary to review the capacity of other staff operating under clinical and other qualified supervision and to train them in higher-level skills of the ART care flow. This could potentially reduce the number of new staff having to be recruited with highest level skills and enhance the availability of existing categories for more complex care. Examples of this kind of skills transfer provided by the ART team during site visits included:

• moving the pharmacist from an operational role to a supervisory role;

• the pharmacy technician filling and dispensing monthly prescriptions to stable antiretroviral (ARV) patients;

• counselling tasks conducted during group sessions, in effect using patients' waiting time for counselling purposes;

• nurses taking over more patient triage (channelling) and patient management tasks (a common practice, given the strong team spirit created in the care team, and already common practice in PHC teams);

• lay counsellors/volunteers/PLWAs on treatment taking over a more prominent role in counselling and other elements of the continuum-of-care spectrum with some supportive supervision and orientation.

**Figure 1:**
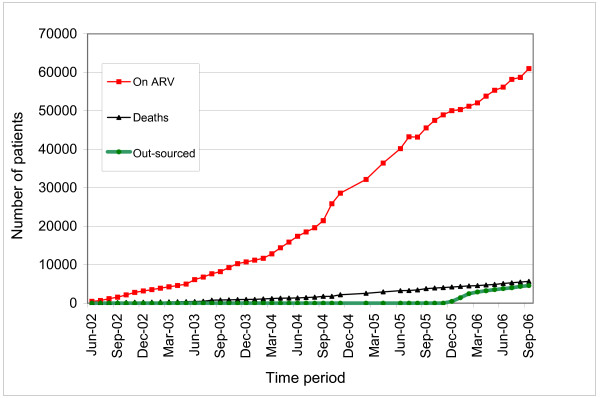
Cumulative number of patients currently on ART in the public and private sector, September 2006 [9].

These proposed measures were subsequently implemented and led to the intended scale up of ART access (see Figure 1, illustrating the trend in increasing numbers of patients on ART, beginning in mid 2004).

### Private sector contributions

Another area reviewed was the potential private sector contribution in the follow-up of stable patients on ART.

### Potential corporate sector contributions

While it was not possible to quantify the treatment capacity of each of these initiatives and more specifically that of ART providers, it can be assumed that company occupational health facilities and programmes could be further investigated for their capacity to take on more patients, thus increasing access opportunities. The example of a mining company based programme with community mobile visits can be viewed online [6]. It would seem that an exploration of examples like this in Botswana could possibly expand the scope of coverage achieved by the corporate sector, with the right high-level political endorsement and contributory funding modes.

### Potential private practice contributions

Private practice is a salient feature of the pluralist Botswana health system, with medical insurance available through employment contracts and discussions were held with between the ministry of health and members of the Botswana Medical Association in order to explore willingness to expand their involvement. In 2003, members of the Botswana Branch of the South African HIV Clinical Society surveyed 100 medical doctors throughout the country in public and private health facilities. Results indicated a high willingness among private practitioners to provide ART on private practice premises (39 out of 40 private practitioners who responded to the survey). If a mechanism could be introduced to fund these services for those who were not covered by health insurance, private practice could potentially contribute substantially to scaling up access to ART, which was estimated to be around 7000 patients per year [7]. In 2004, of the approximately 160 practitioners available throughout the country, 46 had received initial training on ART by participating in the KITSO Botswana training programme. Willingness to share the treatment burden and thus relieve the public sector of patient pressure was ascertained further. In 2004, private practitioners provided ART services to approximately 6000 private patients from their own resources. Opening up this treatment avenue further for a portion of patients currently covered in the public sector could potentially alleviate pressure on the public services. It would broaden access for patients to medically qualified practitioners in line with the treatment model. Furthermore, given that premises were available, costs for further expansion of facilities could also be saved while providing care to more patients. Sharing of drug procurement lines and bulk purchasing arrangements could decrease costs for ARVs and provide efficiencies to government to enlarge the number of persons on ART at a lower drug bill for the nation.

Other options for co-opting private practitioners into public ART care could also be explored by purchasing, for example, private practitioner time in government clinics on a contract basis. It was argued that if, for example, 160 doctors could spend 25% of their time in a public/private partnership, then the current medical officer shortage of 40 in the public sector could be alleviated within a short period. During a national emergency situation, these options could be explored with private practitioners and their associations. It should be borne in mind that private practitioners in Botswana work exclusively on their own premises, and they are not in other employment. Taking on 'contractual' patients for ART from the public sector was therefore in addition to their normal practice workload. The workload was assessed by the Ministry of Health prior to qualifying for participation in the scheme. The main qualifying criteria was successful participation in the KITSO training programme.

The model of care for the public sector ultimately channelled all patients in large numbers to the pharmacist as the only dispensing agent, with the exception of nurses in the PMTCT care flow, thus alleviating the pharmacists' dispensing task.

### Measures taken

After the consultation at the end of 2004 all the suggested options were further explored. The integration of private practitioners into public service delivery could not be an option for the emergency response due to limited space available to accommodate additional staff on the public sector premises among others. As a result the option to outsource patient care to the private sector was chosen. The Ministry of Health started to make preparations to roll out access to private practitioners for new patients eligible for ART as well as those who were stable on ART. The primary aim was to ensure that the waiting period for eligible patients was reduced i.e. patients assessed and found eligible for ART were put on treatment without delays.

To help manage the whole process of outsourcing, the government identified and contracted the services of a disease management company and Associated Fund Agency (AFA) Botswana, a health insurance company. Legally binding provisions were entered to guarantee the smooth functioning of the operations [8]. Measures were undertaken to ensure a system that would allow for maximum access of up to 10 000 patients to the private sector initially, with a view to monitoring the scheme and deciding upon further expansion after one year of operation. The necessary administrative and other arrangements were completed during 2005. Under the agreement, AFA Botswana was to provide fee-for-service reimbursement of patients transferred from the public clinics to the private practitioners. A pilot programme involving 29 private practitioners was initiated in two districts with the highest numbers of eligible patients on the waiting list (Gaborone and Selebi Phikwe). Some 1200 patients stable on antiretroviral were referred within a quarter of a year of operating the scheme. No workload problems were reported from the increase in patient load.

Measures taken to expand public sector access and increase public/private sector collaboration furthered the rapid expansion of service points. Thus, as of September 2006 a total of 60 968 patients had access to ART through the public sector and an additional 4559 were outsourced to the private sector through the collaborative scheme set up in 10 out of a total of 32 ART sites across the country and with BCL. In addition, the private sector provided access to treatment for another 8500 patients through private health insurance, Debswana diamond mining company, Botswana Meat Commission and others.

Furthermore, bottlenecks in access to human resources have been addressed with experience gained from authorizing pharmacy technicians, as well as nurses to dispense and permitting nurses to take over a bigger role in follow up of stable patients on ART. The Integrated Management of Adult and Adolescent Illness approach to expanding access to human resources and services by increased skills transfer is also under implementation, adding to enhanced access to skilled human resources for HIV/AIDS prevention, treatment and care.

### Successes and challenges for out-sourcing

A formal evaluation of the outsourcing initiative is planned for the end of 2007, but a number of lessons, successes and challenges have been observed. The following are some of the successes already acknowledged:

▪ A total of 37 private providers have been contracted to offer ART follow up services. All have government specified qualifications. In addition, two private providers have been contracted to deliver laboratory and diagnostic services;

▪ The reimbursement system is functioning well;

▪ Private providers are involved in system monitoring through monthly reports and this is supported by supervisory visits;

▪ A total of 4559 patients have been referred so far to the private sector;

▪ The private providers refer patients needing welfare services to the public sector, thus the public-private system is well integrated;

▪ A private courier service has been contracted to supply medications to the private sector.

The main challenge experienced so far has been the limited absorptive capacity of the private sector. This has set a limit to the number of patients that could be enrolled. For example in two sites (Selebi Phikwe and Gumare), further enrolment of patients was stopped because the private sector had reached its maximum capacity. In Selebi Phikwe, capacity was further augmented by engaging BCL company as part of the outsourcing. BCL agreed to enrol both its employees and members of the community. There is anecdotal evidence that the outsourcing of ART services is having a real, positive impact on the ground in decreasing patient waiting time and ART clinic congestion. In addition, high patient satisfaction has been observed. The planned year-end evaluation will shed more light on the experience. Figure 1 indicates enrolment progress over the past four years.

## Conclusion

Issues of equitable access in the use of private medical resources come to mind when introducing such a scheme. Expansion of private medical services has been identified as an impediment to ensuring equity in access and fairness in service delivery. In the context of responding to an emergency situation in Botswana, however, the country had to make a critical choice between hiring more medical staff from neighbouring countries with the result of contributing to shortages there, or maximizing the use of existing local resources. By shifting part of the workload from the public sector human resources to the private sector, no economic barriers were created, as access to ARVs remained free. More research, however, will need to be conducted to evaluate the impact of this expansion model across socioeconomic strata of society. What is clear, however, is that shifting nearly 4600 patients into the private sector for treatment and follow up has substantially contributed to reducing the need for additional medical workforce personnel within the Botswana treatment model. By out-sourcing this demand for care, Botswana has effectively reduced its supply need, which would have to come from other countries in the region. The intended coverage goal of 10 000 patients on the out-sourcing model thus actually translates into a regional medical doctor gain for other countries in the order of up to 40 doctors, depending on the frequency and number of visits per year. In a supply restricted environment such as sub-Saharan Africa this is a substantial saving on potential demand-driven losses to other countries.

Another aspect which had been noted by the Manpower Division of the Ministry of Health – as initiatives such as PEPFAR, ACHAP and others unfolded – was an internal 'brain drain' of staff within the country. But this amounted to no more than a couple of dozen staff losses. Given that these human resources stayed in the country and worked in a more focused way on the response to HIV, this was not considered a major loss.

In conclusion, this emergency response to expanding access to human resources rapidly across the available health systems workforce seems to provide a welcome relief for the public sector. In addition, it also prevents the public sector from entering into longer-term staffing commitments that would be difficult to change once the epidemic has come under control and subsides, resulting in less pressure on the health workforce. It will be important to review this experience of private sector cooperation in order to draw conclusions about patient satisfaction, public-private service provider interaction and the lessons that can be extracted for further development. This also demonstrates the potential of using the whole health system (both public and private) to optimize the use of available resources while strengthening the stewardship role to regulate and supervise the different providers.

Engaging the private sector in this way seems a promising way forward in the Botswana context. The applicability of this model to other countries, however, needs to be seen in light of the way private practice is evaluated and regulated in each specific country context. Where dual public-private practice exists as a matter of being tolerated, expansion of access to skilled human resources for a specific service such as ART requires specific policy and regulatory measures.

Study of the mechanism for organizing reimbursement for specific services needed, as is the case in Botswana, may, however, help identify ways of improving access to needed human resources skills by way of contracting.

## Competing interests

The author(s) declare that they have no competing interests.
